# Temporal clustering of gene expression links the metabolic transcription factor HNF4α to the ER stress-dependent gene regulatory network

**DOI:** 10.3389/fgene.2013.00188

**Published:** 2013-09-24

**Authors:** Angela M. Arensdorf, Diane DeZwaan McCabe, Randal J. Kaufman, D. Thomas Rutkowski

**Affiliations:** ^1^Department of Anatomy and Cell Biology, University of Iowa Carver College of MedicineIowa City, IA, USA; ^2^Del E. Webb Neuroscience, Aging and Stem Cell Research Center, Sanford Burnham Medical Research InstituteLa Jolla, CA, USA; ^3^Department of Internal Medicine, University of Iowa Carver College of MedicineIowa City, IA, USA

**Keywords:** ER stress, fatty liver, functional genomics, gene regulatory network, lipid metabolism

## Abstract

The unfolded protein response (UPR) responds to disruption of endoplasmic reticulum (ER) function by initiating signaling cascades that ultimately culminate in extensive transcriptional regulation. Classically, this regulation includes genes encoding ER chaperones, ER-associated degradation factors, and others involved in secretory protein folding and processing, and is carried out by the transcriptional activators that are produced as a consequence of UPR activation. However, up to half of the mRNAs regulated by ER stress are downregulated rather than upregulated, and the mechanisms linking ER stress and UPR activation to mRNA suppression are poorly understood. To begin to address this issue, we used a “bottom-up” approach to study the metabolic gene regulatory network controlled by the UPR in the liver, because ER stress in the liver leads to lipid accumulation, and fatty liver disease is the most common liver disease in the western world. qRT-PCR profiling of mouse liver mRNAs during ER stress revealed that suppression of the transcriptional regulators C/EBPα, PPARα, and PGC-1α preceded lipid accumulation, and was then followed by suppression of mRNAs encoding key enzymes involved in fatty acid oxidation and lipoprotein biogenesis and transport. Mice lacking the ER stress sensor ATF6α, which experience persistent ER stress and profound lipid accumulation during challenge, were then used as the basis for a functional genomics approach that allowed genes to be grouped into distinct expression profiles. This clustering predicted that ER stress would suppress the activity of the metabolic transcriptional regulator HNF4α—a finding subsequently confirmed by chromatin immunopreciptation at the *Cebpa* and *Pgc1a* promoters. Our results establish a framework for hepatic gene regulation during ER stress and suggest that HNF4α occupies the apex of that framework. They also provide a unique resource for the community to further explore the temporal regulation of gene expression during ER stress *in vivo*.

## Introduction

Originally identified as a program for improving protein folding during ER stress, the vertebrate UPR is composed of three separate but functionally overlapping pathways that culminate in transcriptional upregulation of genes involved in ER protein folding and processing (Ron and Walter, 2007). The ER-resident endoribonuclease IRE1, which is conserved among eukaryotes and exists as α [GenBank:NM_023913.2] and β [GenBank:NM_012016.2] paralogs in mammals, catalyzes splicing of *Xbp1* mRNA [GenBank:NM_013842.3 and NM_001271730.1] to remove a 26 base intron and allow for the translation of a transcriptional activator of the bZIP family. The PERK kinase [GenBank:NM_010121.2] is metazoan-specific, and it phosphorylates the translation initiation factor eIF2α [GenBank:NM_001005509.2] when activated, resulting in transient inhibition of protein synthesis but also specific translation of *Atf4* mRNA [GenBank:NM_009716.2] to produce the bZIP transcriptional activator ATF4. Other ER stress-independent eIF2α kinases exist, and phophorylation of eIF2α and the attendant consequences of that event are known as the integrated stress response. ATF6, also metazoan-specific and with α [GenBank:NM_007348.3] and β [GenBank:NM_017406.4] paralogs, is resident to the ER but transits to the Golgi during stress, where it is cleaved by regulated intramembrane proteolysis to liberate an active bZIP transcriptional activator. Together, these bZIPs coordinate enhancement of protein synthesis, degradation, folding, modification, and trafficking through gene regulation.

An oft overlooked feature of UPR activation is that between 20 and 50 percent of regulated genes are actually suppressed by ER stress depending on the conditions, yet much less is known about the mechanisms responsible for this suppression and the physiological consequences thereof. A portion of this suppression can be attributed to regulated IRE1-dependent decay, in which the IRE1 endonuclease degrades ER-associated mRNAs (Hollien and Weissman, 2006). Transcriptional mechanisms for suppression have been identified as well, including direct suppression by the bZIP C/EBP family member CHOP [GenBank:NM_007837.3] (Ron and Habener, 1992), titration of the coactivator CRTC2 [GenBank:NM_028881.2] (Wang et al., 2009), and translational regulation of the suppressive LIP isoform of C/EBPβ [GenBank:NM_009883.3] (Li et al., 2008; Arensdorf and Rutkowski, 2013). Each of these mechanisms was identified through the behavior of target genes, so the extent to which any of them contributes to global gene suppression is not clear.

In the liver, the most evident consequence of ER stress is lipid accumulation (Rutkowski et al., 2008; Yamamoto et al., 2010; Zhang et al., 2011). This lipid accumulation, or steatosis, is accompanied by suppression of a host of genes involved in hepatic lipid metabolic processes, including fatty acid oxidation, lipogenesis, cholesterologenesis, and VLDL production. Given that some of these processes are mutually antagonistic (e.g., fatty acid oxidation and lipogenesis), it seems likely that some are suppressed as primary responses to ER stress, and others as secondary consequences of feedback mechanisms.

Some regulation of hepatic lipid metabolism can be attributed directly to the action of canonical UPR signaling. XBP1 can bind to the promoters and stimulate transcription of lipogenic genes (Lee et al., 2008) and of the ER oxidoreductase PDI [GenBank:NM_001032.2] (Wang et al., 2012), the latter of which stimulates VLDL secretion by virtue of its interaction with the microsomal triglyceride transfer protein (MTTP) [GenBank:NM_001163457.1]. IRE1α can also directly degrade mRNAs encoding lipogenic genes when *Xbp1* is ablated through its regulated IRE1-dependent decay activity (So et al., 2012), thus acting at cross-purposes with its downstream target XBP1. The ER-localized transcription factor CREBH [GenBank:NM_145365.3] also contributes to lipid homeostasis (Zhang et al., 2012), although it is not yet clear whether that function is direct or indirect.

Steatosis arises as a common phenotype in response to ER stress when any of the UPR signaling pathways is ablated, or when UPR signaling is intact but ER protein folding is compromised by deletion of the ER cochaperone p58^IPK^ [GenBank:NM_008929.3] (Rutkowski et al., 2008). This steatosis is likely at least partially caused by impaired secretion of triglyceride-rich VLDL particles from the stressed ER (Ota et al., 2008; Rutkowski et al., 2008; Caviglia et al., 2011) and enhanced uptake (Jo et al., 2013). However, ER stress also elicits extensive alterations in the expression of genes involved in lipid metabolism, and these alterations are more severe and persistent when any branch of the UPR—or when ER protein folding—is compromised (Rutkowski et al., 2008). Thus, these alterations correlate with the development of steatosis, although it is not known which events precede lipid accumulation and which follow as a consequence. That they emerge irrespective of which UPR pathway is ablated argues that most such metabolic genes are not directly regulated by ATF4, ATF6, or XBP1, but by some mechanism or mechanisms that indirectly tie metabolic gene regulation to the ER stress burden. To some degree the dysregulation of lipid metabolism can be attributed to CHOP (Chikka et al., 2013), which is redundantly regulated by each of the three UPR pathways and which is expressed more robustly when stress is more severe—as when the UPR or ER protein folding is disrupted (Rutkowski et al., 2008). However, *Chop*^−/−^ animals are only partially protected from hepatic steatosis during ER stress (Rutkowski et al., 2008), suggesting that other as yet uncovered pathways exist as well.

Given the extensive nature of metabolic gene regulation during ER stress, there likely exists a mechanistic hierarchy of regulation, with some metabolic genes being more proximally connected to UPR pathways and others lying downstream of these initial events. However, the global organization of lipid metabolic gene regulation during ER stress has not been studied. Thus, our goal in this work was to begin to decipher the structure of ER stress-mediated metabolic gene regulation by establishing the temporal progression of such events in the mouse liver, and to infer hierarchical relationships using a functional genomics approach, based upon the behavior of coordinately regulated groups of genes in wild-type mice vs. mice lacking the ER stress sensor ATF6α.

## Materials and methods

### Animal experiments

All protocols for animal use were reviewed and approved by the University Committee on Use and Care of Animals at the University of Iowa or the University of Michigan. Animals were bred in house, and were fed standard rodent chow and housed in a controlled pathogen free environment with 12 h light and dark cycles. Animals used were of varying ages and genders, with control and experimental groups having similar composition. Animals were fasted for 4 h prior to sacrifice, which was carried out in daytime hours.

### Lipid analysis

ADRP immunostaining was as described (Chikka et al., 2013). For the trigylceride assay, a 100 mg piece of liver was homogenized in 1 mL of ice cold extraction buffer (1 mM Tris pH 7.6, 1 mM EGTA, 1 mM MgCl_2_) containing protease inhibitors. A 200 μ L aliquot of the homogenate was placed into a new 1.5 mL tube on ice, and an additional aliquot was set aside to determine the protein concentration of each sample. A 750 μL aliquot of a Chloroform:Methanol mixture (1:2 ratio) was added to the 200 μL homogenate sample and vortexed vigorously for 15 s. The samples were incubated at room temperature for 1 h, with the samples vortexed every 15 min. Following the hour incubation, 250 μL of Chloroform was added to each sample and vortexed for 15 s, then incubated at room temperature for 15 min. Two hundred μL of distilled water was then added to the sample and vortexed as above. The samples were centrifuged at 5000 rpm for 10 min, and the bottom organic layer was collected and placed in a fresh tube. The sample was evaporated under nitrogen gas, and the remaining lipids were dissolved in 200 μL of isopropanol. From these samples, the triglyceride levels were determined using the Infinity Triglycerides Reagent (Thermo Scientific, TR22421) per manufacturer's instructions. The Cayman triglyceride standard (Cayman Chemical, 10010509) was used to generate a standard curve. Oil Red O staining was as described (Rutkowski et al., 2008).

### RNA analysis

The 8 h microarray has been published (Rutkowski et al., 2008). For the 34 h microarray, mice were injected with 1 mg/kg TM, and mRNA was prepared from isolated livers and analyzed by Affymetrix microarray in exactly the same way. The NCBI GEO accession number for both arrays is GSE48939. Expression categories for each probeset are provided in Table S1. RT-PCR and qRT-PCR analysis, including validation of all primer sets, was as previously described (Rutkowski et al., 2006; Tyra et al., 2012), except that gene expression was normalized against the average expression of two housekeeping genes (*Btf3* and *Ppia*) rather than only one. Primer sequences can be found in (Rutkowski et al., 2006, 2008; Wu et al., 2007; Tyra et al., 2012) and Table S2.

### Bioinformatic analysis

Pathway enrichment analysis was performed using FunNet software (Prifti et al., 2008). Data represent GO biological process annotations with a decorrelated enrichment computation and a 5% false discovery rate correction. Transcription factor binding site analysis was performed using oPOSSUM software (Ho Sui et al., 2005). Data represent a single site analysis of vertebrate transcription factor binding sites within 2000 base pairs upstream or downstream of the transcription start site. The results of this analysis were visualized using Cytoscape software. The network was limited to genes with GO annotations involved in lipid metabolism and transport using the BiNGO plug-in (Maere et al., 2005).

### Chromatin immunoprecipitation

Livers were isolated from 6–8 week-old mice and prepared for ChIP using the ChIP Tissue Chromatin Shearing Kit with SDS (Covaris). Samples were sonicated using a S220 focused ultrasonicator (Covaris) to produce DNA bands between 100 and 1000 bp. Following sonication, the immunoprecipiation was carried out as described in (Arensdorf and Rutkowski, 2013) using HNF4α antiserum (H-171, Santa Cruz) or non-specific IgG (12-370, Millipore).

## Results and discussion

### Suppression of metabolic transcriptional master regulators precedes lipid accumulation during ER stress

Lipid accumulation can be induced in the liver by exposure to the inhibitor of N-linked glycosylation tunicamycin (TM) or the proteasomal inhibitor bortezomib, or by overexpression of a misfolded ER client protein such as coagulation Factor VIII [GenBank:NM_001161373.1] (Rutkowski et al., 2008; Zhang et al., 2011; Chikka et al., 2013). Each of these treatments activates either the UPR or integrated stress response and each leads to qualitiatively similar changes in the expression of key metabolic genes in the liver. These genes include both transcription factors and cofactors involved in controlling metabolism as well as the downstream targets of these factors that encode the key functional enzymes involved in lipid catabolism, anabolism, storage, and secretion (Rutkowski et al., 2008).

**Figure 1:**
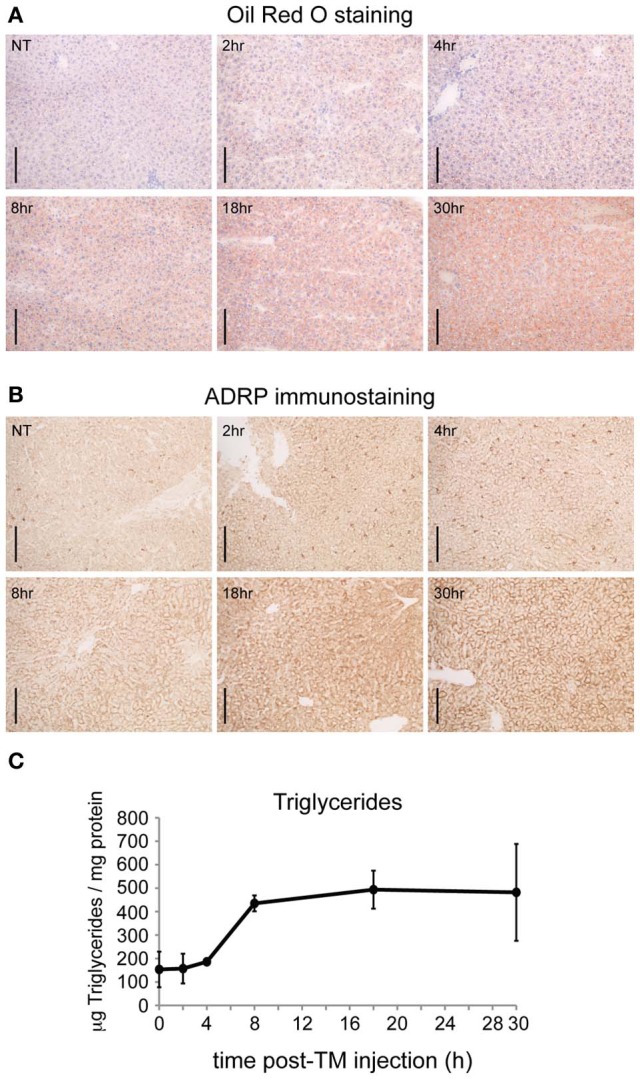
**ER stress causes substantial hepatic lipid accumulation within 8 h. (A)** C57BL/6J mice were challenged with 1 mg/kg TM for the indicated times, livers were frozen in OCT, and lipids were stained with Oil Red O. Scale bar = 50 μm. **(B)** Same as **(A)**, except lipid content was assessed by immunohistochemical staining for the lipid droplet marker protein ADRP. Scale bar = 50 μm. **(C)** Same as **(A)**, except triglyceride content was measured by colorimetric assay after extraction of neutral lipids. *p* < 0.001 by One-Way ANOVA. *n* = 3 samples per time point. Error bars here and elsewhere show means ± SDM.

The temporal organization of these gene regulatory changes prior and subsequent to the onset of lipid accumulation has not been analyzed. It is likely that the earliest of these regulated events are directly mechanistically connected to the UPR and/or integrated stress response. Thus, we examined the time course of hepatic lipid accumulation in wild-type mice in response to TM, which is a more robust inducer of ER stress and steatosis than other stimuli (Chikka et al., 2013). We monitored lipid accumulation by three distinct criteria: accumulation of Oil Red O in lipid droplets of fresh frozen liver sections (Figure 1A); immunohistochemical detection of the lipid-droplet associated protein adipophilin (ADRP) [GenBank:NM_007408.3] (Figure 1B); and direct biochemical assessment of hepatic triglyceride levels (Figure 1C). Results from all three assays were similar: hepatic lipid content increased most substantially at the 8 h time point. Therefore, the key genetic regulatory events responsible for altering lipid metabolism are likely to occur prior to 8 h, while those that occur later than 8 h after challenge are more likely secondary effects that contribute to lipid disruption tangentially, if at all.

**Figure 2:**
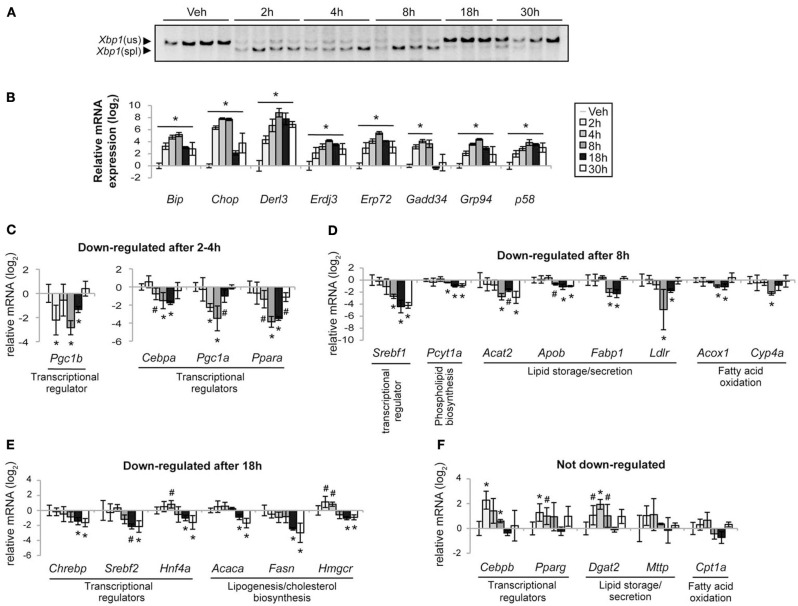
**Staggered suppression of metabolic genes during ER stress. (A)** The spliced (spl) and unspliced (us) forms of *Xbp1* mRNA were detected by RT-PCR of total RNA isolated from the livers of mice treated with vehicle (veh) or 1 mg/kg TM for the indicated times. Each lane shows a separate animal. Image is shown in black-to-white inverted form for greater visual clarity. **(B)** Expression of the indicated UPR target genes was determined by qRT-PCR from the animals shown in **(A)**, with *Btf3* and *Ppia* used as normalizing controls. Expression here and in subsequent figures is given on a log_2_ scale relative to the vehicle-treated condition. Here and elsewhere unless noted: ^*^p < 0.05; ^#^*p* < 0.1 by two-tailed student's *t*-test. **(C–F)** Expression of metabolic genes was assessed by qRT-PCR as in **(B)**, and genes were grouped according to the time point at which downregulation (*p* < 0.1) was first observed. The process in which each gene participates is listed.

Next, we examined the timing of UPR activation and of the regulation of metabolic genes. TM led to maximal IRE1α-dependent splicing of *Xbp1* mRNA within 2 h; this splicing persisted through 8 h but was diminished at later time points (Figure 2A). Likewise, every UPR-regulated target gene was significantly upregulated by 2 h, peaked at 4–8 h, and diminished thereafter (Figure 2B). These genes depend to varying extents on activity of each of the three UPR pathways (Harding et al., 2003; Lee et al., 2003; Wu et al., 2007). That they are uniformly upregulated by 2 h suggests that each of the three canonical UPR-regulated transcriptional activators—ATF6α, ATF4, and XBP1—is functionally active by this very early time point.

We then analyzed expression of genes involved in lipid metabolism, including both transcriptional regulators and key rate-limiting metabolic enzymes. We grouped the genes according to the time point at which they were first down-regulated. We immediately observed that, in contrast to conventional UPR target genes, the regulation of metabolic genes occurred in stages. The earliest group regulated included select transcription factors and cofactors. The coregulator *Pgc1b* [GenBank:NM_133249.2] was downregulated by 2 h although not at 4 h (Figure 2C), making the true timing of its suppression ambiguous. In contrast, by 4 h the coregulator *Pgc1a* [GenBank:NM_008904.2] and the transcriptional activators *Cebpa* [GenBank:NM_007678.3] and *Ppara* [GenBank:NM_001113418.1] were suppressed (Figure 2C). PGC-1β contributes to lipogenesis, fatty acid oxidation, and VLDL production and secretion (Lee et al., 2003; Lin et al., 2003, 2005a,b; Wolfrum and Stoffel, 2006), and PGC-1α contributes to the latter two of these processes (Louet et al., 2002; Lin et al., 2005a; Rhee et al., 2006). C/EBPα has general roles in energy homeostasis including lipid and glucose metabolism, while PPARα is a master regulator of fatty acid oxidation (Desvergne et al., 2006).

The genes downregulated at later times—i.e., after the onset of pronounced lipid accumulation—included both transcriptional regulators and downstream genes encoding rate-limiting enzymes in metabolic processes, as indicated in Figures 2D–F. These results suggest that PGC-1α, C/EBPα, and PPARα (and possibly PGC-1β) are most likely the first lipid metabolic genes regulated by ER stress. Further, the processes downstream of these factors—namely, fatty acid oxidation and lipoprotein biogenesis and transport—are the first affected via gene regulatory mechanisms. In addition, because lipogenic genes are not altered until much later (18 h), they suggest that inhibition of lipogenesis is a secondary consequence of ER stress, perhaps occurring as a result of negative feedback when lipids begin to accumulate. In addition, even though XBP1 splicing is induced by ER stress, we found no evidence that lipogenic gene expression was *stimulated* under these conditions.

### Deletion of ATF6α exacerbates metabolic gene suppression

**Figure 3:**
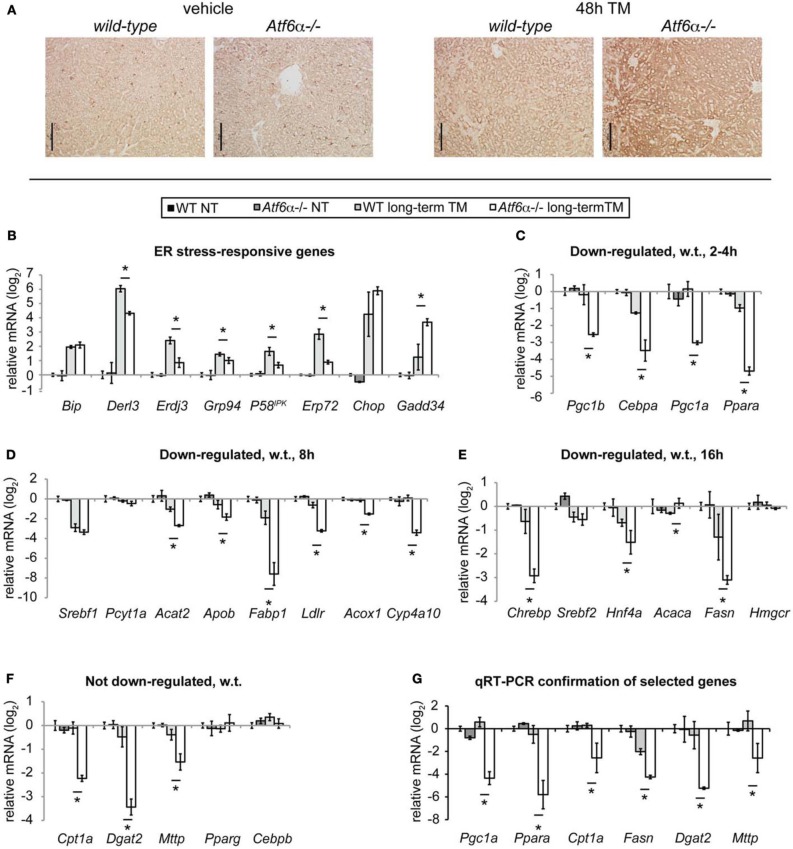
**Loss of *Atf6*α exacerbates steatosis and long-term metabolic gene suppression. (A)** Wild-type or *Atf6*α^−/−^ mice were challenged with 1 mg/kg TM or vehicle for 48 h, and ADRP immunostaining was carried out as in Figure 1. Scale bar = 50 μm. **(B–F)** Wild-type or *Atf6*α^−/−^ mice were challenged with 1 mg/kg TM or vehicle for 34 h, and global mRNA expression was assessed by Affymetrix microarray. Array-determined expression of the indicated genes is shown. Genes were arranged in the same groupings as in Figure 2. Statistical significance was calculated by two-tailed student's *t*-test, comparing expression in TM-treated *Atf6*α^−/−^ animals against TM-treated wild-type animals. *n* = 3 animals per group. **(G)** Mice were treated with TM or vehicle for 48 h as in **(A)**, and expression of the indicated genes was determined by qRT-PCR. Significance was determined as in **(B–F)**.

Mice lacking ATF6α, while otherwise apparently normal and healthy, exhibit dramatic steatosis upon challenge with TM (Rutkowski et al., 2008; Yamamoto et al., 2010). This phenotype is illustrated by ADRP staining in Figure 3A; it arises from an inability to restore ER homeostasis upon challenge. Therefore, we reasoned that these mice could be used to expose the stress-regulated gene expression changes that truly underlie lipid dysregulation, since those changes should be amplified in *Atf6*α^−/−^ animals. A second benefit of these animals is that they can be used to identify truly stress-responsive expression changes; those that result from ER stress-independent properties of TM would not be expected to differ between wild-type and *Atf6*α^−/−^ animals, since ATF6α is not thought to act outside the context of ER stress, and its deletion does not alter the apparent pharmacological activity of TM (Rutkowski et al., 2008).

We approached this goal by challenging wild-type and *Atf6*α^−/−^ animals for an extended period (34 h) with TM, and then profiling global gene expression by microarray. We chose this approach in part because we had previously used microarray profiling to characterize gene expression in these same mouse strains following 8 h of TM challenge (Rutkowski et al., 2008). Conducting a second microarray study with an identical gene chip at a later time point would provide us with the unique opportunity to determine, for every gene on the array, its expression in two different genotypes, under two different treatment regimens (vehicle and TM) at two different times.

By having 8 distinct combinations of experimental conditions, we anticipated that we could begin to identify clusters of coordinately regulated genes, including those metabolic genes most responsive to ER stress. This relied upon the assumption that genes which are part of a common functional pathway (in this case, lipid metabolism) should be regulated similarly. This approach has been used to great effect in yeast, and more recently in cultured mammalian cells, to expose previously hidden components of the secretory apparatus and other pathways of interest (Schuldiner and Weissman, 2013). Here, our goal was to infer hidden transcriptional regulators using temporal patterns of gene expression as an output.

From the 34 h microarray, we examined the expression of the UPR and metabolic genes shown in Figure 2. Consistent with the role of ATF6α in contributing to chaperone expression (Wu et al., 2007; Yamamoto et al., 2007), most ER chaperones and cochaperones known to be regulated by ATF6α, such as *Derl3* [GenBank:NM_024440.2], *Erdj3* [GenBank:NM_001190804.1], *Grp94* [GenBank:NM_011631.1], *p*58^IPK^, and *Erp72* [GenBank:NM_009787.2], were not upregulated to as great an extent in *Atf*6α^−/−^ animals as in wild-type animals (Figure 3B). Conversely, expression of *Gadd34* [GenBank:NM_008654.2], which largely depends upon the PERK axis of the UPR (Marciniak et al., 2004), was elevated in *Atf*6α^−/−^ mice, consistent with persistent ER stress and activation of the other limbs of the UPR.

Amongst the metabolic genes examined, none was upregulated by ER stress in either genotype at 34 h. Two genes—the lipogenic and cholesterologenic regulators *Srebf1* [GenBank:NM_011480.3] and *Srebf2* [GenBank:NM_033218.1]—were equally downregulated in both genotypes (Figures 3D,E). However, the large majority of genes were expressed at normal or near-normal levels in wild-type animals, but deeply suppressed in *Atf*6α^−/−^ animals (Figures 3C–F). qRT-PCR profiling of a sampling of these genes from an independent experiment confirmed these findings (Figure 3G). These data are consistent with the idea that acute ER stress inhibits fatty acid oxidation and lipoprotein biogenesis at the level of gene expression, and does not stimulate lipogenic gene expression.

### Functionally related gene groups cluster temporally

Having these 8 distinct experimental conditions enabled us to compare the global behavior of hepatic gene expression in response to ER stress at early vs. late times in normal animals vs. those with a compromised UPR. A heatmap showing TM-regulated genes makes two observations clear: First, gene expression differences between the two genotypes were far more extensive at 34 h than at 8 h. Second, at 34 h, many genes have returned to normal or near-normal expression in wild-type animals, but have remained regulated, or become even more regulated in the same direction, in *Atf*6α^−/−^ animals.

Each gene was then categorized based on whether it was upregulated, downregulated, or unchanged by ER stress in wild-type and *Atf*6α^−/−^ animals after 8 or 34 h of stress. Thus, a gene could fall into any of 81 (3 × 3 × 3 × 3) expression profiles. The assignments for all probesets are provided in Table S1. We were able to analyze the genes in this way in part because very few genes differed in their basal (i.e., unstressed) expression between genotypes; almost all genotype-dependent changes in expression were caused by TM, so basal expression differences were not confounding (Rutkowski et al., 2008).

**Figure 4:**
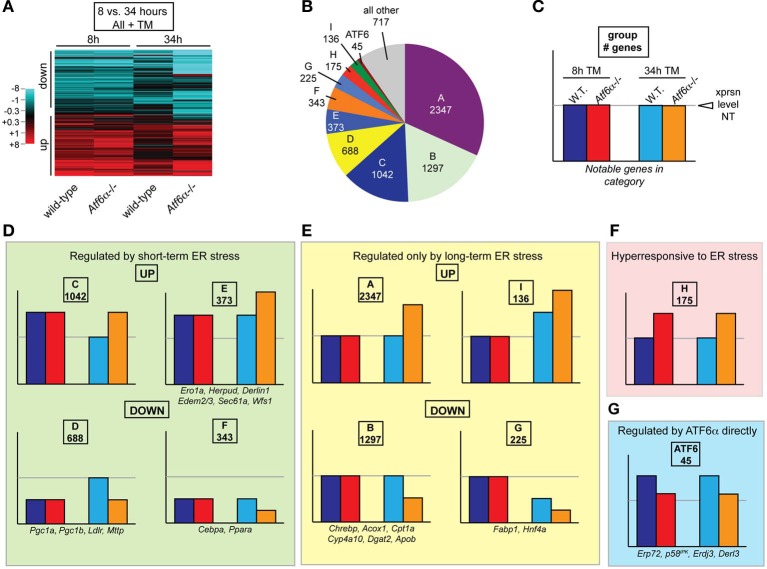
**Clustering of genes according to temporal regulation in wild-type and *Atf*6α^−/−^ animals. (A)** The expression of every probeset on the Affymetrix microarray described in Figure 3 was aggregated with expression data from a previously published identical array comparing gene expression in wild-type and *Atf*6α^−/−^ animals 8 h after challenge with vehicle or 2 mg/kg TM (Rutkowski et al., 2008). For each time point, expression was determined using a log_2_ scale relative to vehicle-treated wild-type animals at that time point. Only probesets showing significant (*p* < 0.05) expression differences (1.5-fold, or ±0.58 on the log_2_ scale) in one or more of the four of the four conditions (wild-type or *Atf*6α^−/−^ at 8 or 34 h) are shown by heatmap, which accounted for ~7000 of the ~45,000 probesets on the array. Extent of up- or downregulation is shown by intensity of red or blue coloration, respectively. Each column depicts expression level averaged among the three animals per group. **(B,C)** Every probeset on the array was characterized by its expression in the following four ways, with differences defined as > 1.5-fold, *p* < 0.05: (1) up, down, or unchanged in wild-type TM-treated animals at 8 h relative to vehicle-treated wild-type; (2) up, down, or unchanged in *Atf*6α^−/−^ TM-treated animals at 8 h relative to TM-treated wild-type; (3) same as (1) but 34 h; and (4) same as (2) but 34 h. The genes that showed a difference by criterion (4) were broken down into groups based on their behavior with respect to these criteria, and the number of genes in the nine most populated groups is shown in **(B)**. The group of genes that were upregulated by ER stress in wild-type animals at both time points, but were less upregulated in *Atf*6α^−/−^ animals—i.e., genes that could be understood as directly ATF6α-dependent—is also accounted for. **(C)** provides a key for illustration of gene expression patterns. **(D–G)** Expression pattern for each of the gene groups shown in **(B)**. These include genes shown in Figure 2 (those that did not fall into one of these groups are illustrated in Figure S1) as well as genes involved in ER protein processing found in Group E. For genes represented by more than one probeset, the behavior most commonly represented and/or most consistent with qRT-PCR data is shown.

Because *Atf*6α^−/−^ animals became more steatotic than wild-type animals, genes that were not differentially expressed between the two genotypes upon 34 h of TM treatment were not pursued further, as these were unlikely to be major contributors to the steatotic phenotype. Although 54 remaining combinations of expression were possible (3 × 3 × 3 × 2), 90 percent of the genes fell into only 9 categories (A–I, Figure 4B). Each category of genes was then given a graphical representation of the expression pattern of its members (Figures 4C–G). The two most populated groups of genes (Groups A and B) were those that were unaffected by stress at 8 h in either genotype, or in wild-type animals at 34 h, but were up- or down-regulated, respectively, at 34 h in *Atf*6α^−/−^ animals (Figure 4E). Among the metabolic genes depicted in Figures 2, 3, a number of these populate Group B, and they include genes encoding rate-limiting enzymes in each pathway of fatty acid oxidation—*Acox1*, *Cpt1a*, and *Cyp4a10* [GenBank:NM_015729.3, NM_013495.2, and NM_010011.3], which control peroxisomal, mitochondrial, and microsomal oxidation, respectively. As the expression of these genes is not altered until the later timepoint, they are unlikely to be proximally connected to the UPR, but more likely represent indirect effects—for example, of suppression of PPARα. The next two most populated groups (Groups C and D) included those genes that were either up- or down-regulated early in both genotypes, and whose expression returned to normal levels in wild-type animals by 34 h but which remained regulated in *Atf*6α^−/−^ animals. Among metabolic genes, Group D included the coregulators *Pgc1a* and *Pgc1b*. Closely related to these groups were Groups E and F, which included genes that were up- or downregulated early in both genotypes, and for which this regulation was enhanced in *Atf*6α^−/−^ animals at 34 h. The other two rapidly regulated metabolic genes, *Cebpa* and *Ppara*, were found in Group F.

The lipogenic genes *Fasn* [GenBank:NM_007988.3] and *Acaca* [GenBank:NM_133360.2] and the cholesterologenic genes *Acat2* [GenBank:NM_009338.3] and *Hmgcr* [GenBank:NM_008255.2] showed no evidence of upregulation in the 8 or 34 h array data, and *Fasn* and *Acat2* were actually suppressed, as were *Srebf1* and *Srebf2* themselves (Figure S1). While lipogenesis can be stimulated by non-transcriptional mechanisms—most notably processing of SREBP-1c and SREBP-2—this processing would result in stimulation of their downstream target genes, which is not evidenced here.

Groups D and F were of the most interest to us because they represented those genes most likely to be proximally mechanistically connected to UPR signaling, since they were regulated rapidly by ER stress and since their long-term expression coincided with the persistent ER stress and worsening lipid accumulation seen in *Atf*6α^−/−^ animals. Group F in particular was noteworthy because its counterpart cohort of upregulated genes—Group E—included a number of genes known to be direct targets of UPR transcription factors and which are thus proximally connected to UPR signaling (Figure 2D).

Also supporting the validity of the approach was the group of genes that were upregulated by ER stress in wild-type animals at both time points, but that were not as upregulated in *Atf*6α^−/−^ animals at both time points (Figure 4G). These genes would fit the expected profile of direct targets of ATF6α. While there were relatively few genes in this group, they included those already described as direct ATF6α targets, including *Erp72*, *p*58^IPK^, *Erdj3*, and *Derl3* (Wu et al., 2007; Yamamoto et al., 2007). This finding supports the idea that coregulated genes can be discriminated based on their expression profile in this setup.

**Figure 5:**
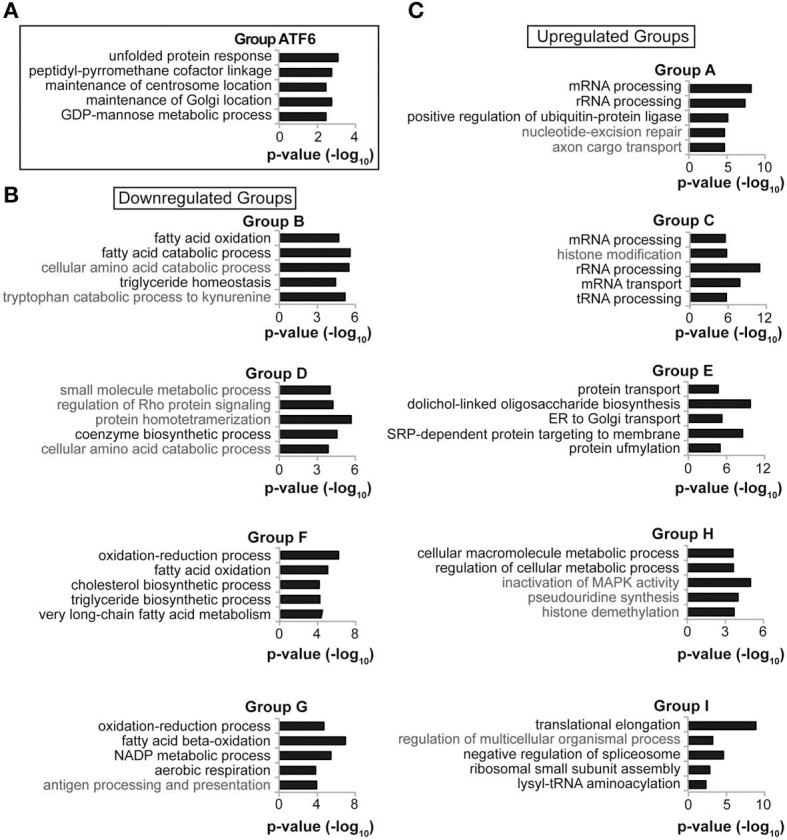
**Functionally related genes cluster by temporal regulation. (A–C)** Each of the gene groups in Figure 4 was subjected to Gene Ontology pathway analysis using FunNet. The top seven most significant pathway enrichments were then reordered by the number of genes from that group that were pathway “hits,” with pathways having the most “hits” listed higher. For reasons of space, five of these seven pathways from each group are shown. In no case was a lipid metabolism process enriched among the upregulated gene groups. In **(B)**, pathways relevant to lipid metabolism are listed in black, and other pathways in gray. In **(C)**, pathways relevant to protein processing are listed in black.

This idea was reinforced by pathway analysis. The genes from each of the ten groups (A–I and ATF6) were analyzed for functional enrichments of Gene Ontology (GO) pathways using FunNet (Prifti et al., 2008). As proof-of-concept, pathway analysis of Group ATF6 genes yielded “unfolded protein response” as the most significantly enriched process, which would be expected of a group encompassing ATF6α direct targets (Figure 5A). Genes representing processes relevant to lipid metabolism were enriched in all of the downregulated groups—B, D, F, and G—but not in the upregulated groups A, C, E, H, and I (Figures 5B,C). Conversely, each of the upregulated groups was enriched in genes representing pathways relevant to protein synthesis, trafficking, and degradation. UPR activation during ER stress is known to transcriptionally augment the cellular protein biogenesis machinery through the action of the major UPR-regulated transcriptional activators XBP1, ATF4, and ATF6 (Harding et al., 2003; Lee et al., 2003; Wu et al., 2007). This pathway analysis thus suggests that, at least in the liver, suppression of genes involved in lipid metabolism represents a concerted focus of UPR activation.

### Functional genomic analysis identifies HNF4α as a proximal regulator of metabolic gene expression during ER stress

**Figure 6:**
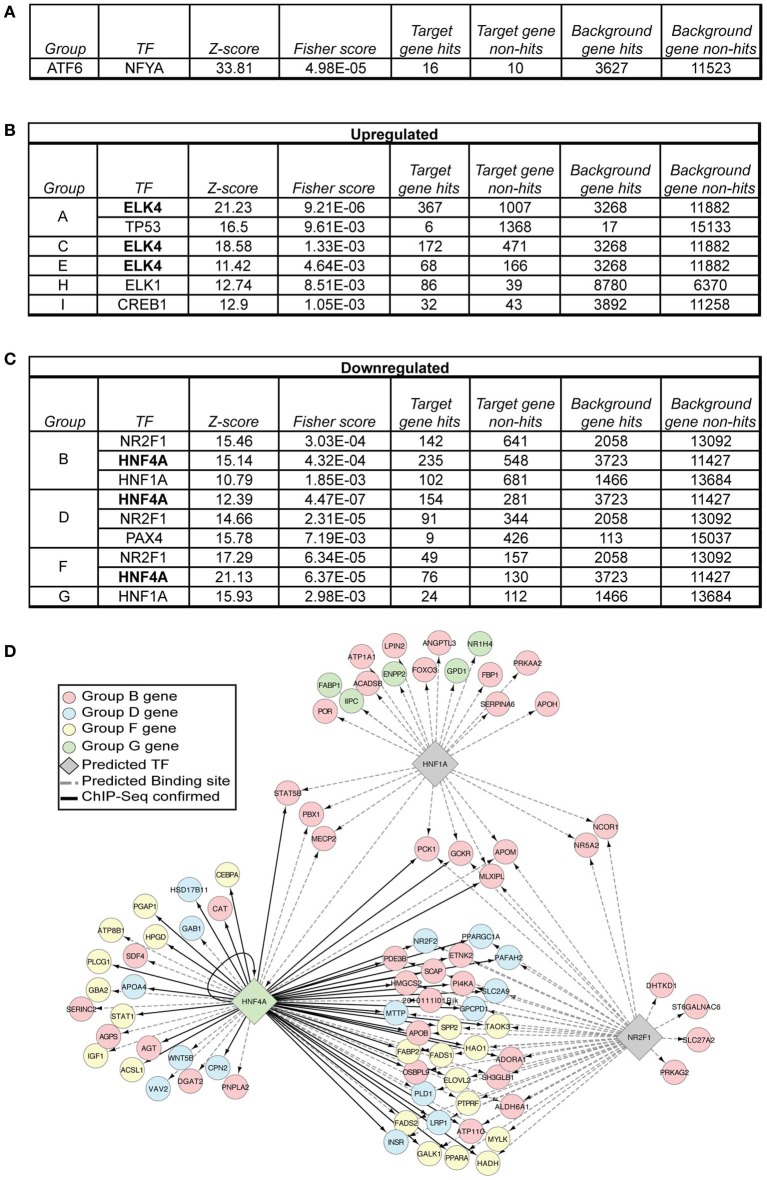
**Transcription factor prediction implicates ELK4, HNF1α, NR2F1, and HNF4α as hidden regulatory nodes in hepatic stress-dependent gene regulation. (A–C)** Each of the gene groups in Figure 4 was subjected to oPOSSUM single-site analysis, which searches regulatory regions (in this case, ±2000 bp from the transcriptional start site) for potential binding sites of transcription factors identified in the JASPAR CORE database. The results were limited to a *Z*-score > 10 and a Fisher score < 0.01. **(D)** The data from **(C)** were visualized using the BINGO plug-in for Cytoscape software, considering only genes relevant to lipid metabolism as annotated from GO analysis. oPOSSUM-predicted binding sites are shown using dashed lines, while genes with confirmed HNF4α-binding sites are shown by solid lines.

We wished to harness the statistical power of our microarray comparisons in order to predict regulatory transcription factors whose activity was altered by ER stress; these would be the most likely to be proximally mechanistically connected to the UPR. To accomplish this, we subjected the genes in each group to analysis using oPOSSUM (Ho Sui et al., 2005), which searches the promoter/enhancer region of each gene for consensus transcription factor binding sites from the JASPAR CORE database. Establishing the validity of this approach, the genes in Group ATF6 yielded the transcription factor NFYA [GenBank:NM_001110832.1] as the sole statistically significant hit (Figure 6A). ATF6α is known to dimerize with NFYA to regulate transcription from promoters containing ERSE and ERSE II elements (Yoshida et al., 2000, 2001; Kokame et al., 2001). NFYA was not enriched in any other group, underscoring the specificity of the algorithm.

Because genes involved in lipid metabolism were found in groups B, D, F, and G, we sought hits found in those groups and no others, with a particular emphasis on groups D and F, since these groups contain the early-responding genes. Remarkably, binding sites for two transcription factors—HNF4α and NR2F1 [GenBank:NM_010151.2]—were enriched in Groups B, D, and F but not in any other group (Figures 6B,C). These factors have similar consensus binding sites (Kimura et al., 1993; Ellrott et al., 2002; Schmidt et al., 2010), explaining why they segregate together in this analysis. This finding suggests that the activity of these two transcriptional activators is altered during ER stress, since genes containing binding sites for these factors are downregulated by ER stress, and their downregulation is exacerbated by the ongoing stress seen in *Atf*6α^−/−^ mice. Conversely, groups A, C, and E were enriched for genes with potential binding sites for ELK4, also known as SRF Accessory Protein (SAP)-1 [GenBank:NM_007923.2].

Group I, encompassing genes upregulated by long-term ER stress in wild-type animals and further upregulated in *Atf*6α^−/−^ animals, was enriched for binding by CREB1 [GenBank:NM_001037726.1], which shares a consensus binding sequence with the ER-localized transcription factor CREBH (Zhang et al., 2006). CREBH is activated by proteolysis induced by, among other stimuli, ER stress, and it regulates expression of acute phase response genes and metabolic genes (Zhang et al., 2006, 2012; Luebke-Wheeler et al., 2008). Accordingly, the genes in Group I have a profile expected for CREBH targets—namely that they are upregulated by ER stress, and further upregulated in *Atf*6α^−/−^ animals, in which ER stress is exacerbated. However, lipid metabolic genes were not enriched in Group I, and the CREB1 binding site was not enriched in the groups containing lipid metabolic genes. In addition, other than the gene encoding serum amyloid P-component (*Apcs*) [GenBank:NM_011318.2], most genes encoding acute phase response proteins (SAA proteins, CRP, coagulation and clotting proteins, etc.) were not significantly upregulated at either time point in either genotype, suggesting that CREBH activity during *bona fide* ER stress (as opposed to endotoxin, pro-inflammatory cytokines, or other stimuli) might be minimal, and the genes in Group I might instead be regulated by another factor from the CREB family.

ELK4 is part of the Ternary Complex Factor (TCF) family of transcription factors that interact with Serum Response Factor (SRF) (Dalton and Treisman, 1992). A role for ELK4 in hepatic gene expression has not been described, nor has ELK4 been directly linked to ER stress. ELK4 has been shown to be activated by JNK-dependent phosphorylation (Janknecht and Hunter, 1997), and JNK [GenBank:NM_016700.4] is activated by ER stress (Urano et al., 2000). Thus, we speculate that ELK4 activity might be regulated during ER stress in the liver by JNK or other MAP kinases to promote expression of genes in groups A, C, and E. NR2F1, also known as COUP-TF, is an orphan receptor. Deletion leads to perinatal lethality with extensive dysregulation of neuronal differentiation (Qiu et al., 1997). Its function in the liver is less clear, although it has been shown to coactivate transcription synergistically with HNF4α (Ktistaki and Talianidis, 1997; Yanai et al., 1999).

HNF4α is expressed most strongly in the liver, intestine, kidney, and pancreas. Deletion is lethal during gastrulation (Chen et al., 1994). Mice with a liver-specific deletion of HNF4α develop steatosis concomitant with impaired *ApoB* and *Mttp* expression and VLDL production (Hayhurst et al., 2001). Hepatic knockdown of HNF4α by adenoviral delivery resulted in steatosis and in impaired VLDL production, along with essentially uniformly diminished expression of a host of genes involved in lipid metabolism, including many of those reported here (Yin et al., 2011). Thus, loss of HNF4α phenocopies many of the lipid metabolic genetic changes that are induced by ER stress. Overexpression of HNF4α in primary hepatocytes resulted in upregulation of many of these same genes, though not of *Srebf1* nor *Srebf2* (Yin et al., 2011). This exception is notable because *Srebf1* and *Srebf2* are conspicuous among the metabolic genes analyzed here in the fact that their downregulation is *not* exacerbated at 34 h in *Atf*6α^−/−^ mice (Figures 3D,E). HNF4α binding sites in the mouse liver genome have been characterized by ChIP-seq (Schmidt et al., 2010), and genes in Groups B, D, and F are confirmed HNF4α targets (Figure 6D).

**Figure 7:**
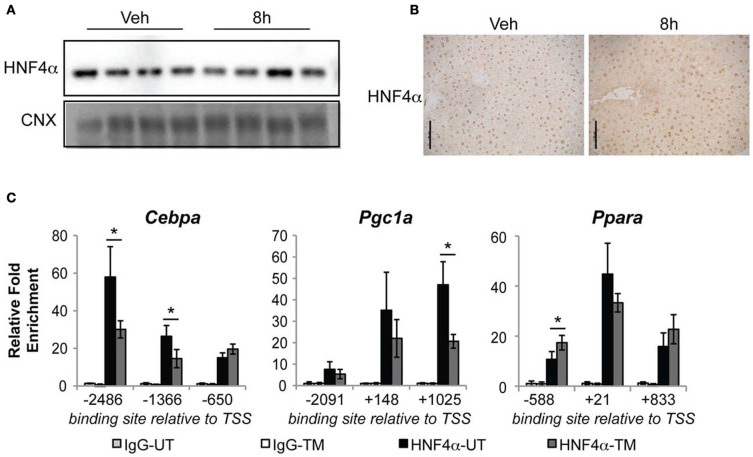
**Diminished HNF4α binding at *Cebpa* and *Pgc1a* promoters during ER stress. (A,B)** Wild-type mice were challenged with 1 mg/kg TM for 8 h, and expression of HNF4α was assessed by immunoblot **(A)** or immunohistochemistry **(B)**. Scale bar = 50 μm. **(C)** HNF4α binding to the regulatory regions of the indicated genes was assessed by chromatin-IP. Regions are given relative to the transcriptional start site, and correspond to regions identified by ChIP-seq analysis (Schmidt et al., 2010). *n* = 3–4 animals per group. Typical recovery of genomic material in samples containing HNF4α antibody was in the range of 0.1–1 percent of total input. ^*^p < 0.05 by *t*-test.

Our results, together with the known activity of HNF4α, predict that ER stress leads to diminished activity of HNF4α, and that this occurs as an early event in metabolic gene regulation by ER stress. To test this prediction, we analyzed the binding of HNF4α to ChIP-seq-defined sites in the promoter/enhancer regions of three of the four earliest regulated metabolic genes—the transcription regulators *Cebpa*, *Pgc1a*, and *Ppara* (the proximal *Pgc1b* promoter/enhancer did not contain a predicted or validated HNF4α binding site). We found that treatment of animals with TM for 8 h did not change either the total expression (Figure 7A) or nuclear localization (Figure 7B) of HNF4α. Yet, consistent with our prediction, we found that HNF4α chromatin binding decreased significantly at several sites in the *Cebpa* and *Pgc1a* promoters upon treatment of animals with TM (Figure 7C). This diminishment was not uniform; several sites within the three promoters showed unaltered HNF4α binding (Figure 7A). Together, these findings suggest that ER stress reduces the activity of HNF4α at specific sites in the genome through a mechanism that is independent of the HNF4α expression level.

## Conclusions

The hepatic lipid dysregulation that is elicited by ER stress is accompanied by sweeping alterations to the expression of genes involved in the process. With few exceptions, these genes are downregulated by ER stress, and encompass numerous metabolic pathways including fatty acid oxidation, lipogenesis, triglyceride storage and secretion, and phospholipid synthesis. The downregulated genes include both those encoding the key enzymes of each of these processes as well as the upstream transcription factors that regulate them. However, it would be unlikely that these genes would be directly suppressed by canonical UPR-regulated transcription factors, since these are, for the most part, transcriptional activators rather than repressors. Further, ablation of the ATF6α pathway of the UPR compromises recovery from ER stress and leads to an exacerbated steatotic phenotype, yet ATF6α does not directly act upon genes involved in lipid metabolism—no such genes populate either group ATF6 or its downregulated converse. Therefore, other mechanisms linking UPR activation to metabolic gene regulation must be at work. The extensive nature of metabolic gene regulation during ER stress also suggests a hierarchical organization of gene regulatory events, with most genes regulated as an indirect consequence of earlier events. Such an organization predicted that the temporal ordering of gene regulation could be used to identify the transcriptional events most proximal to UPR signaling.

**Figure 8:**
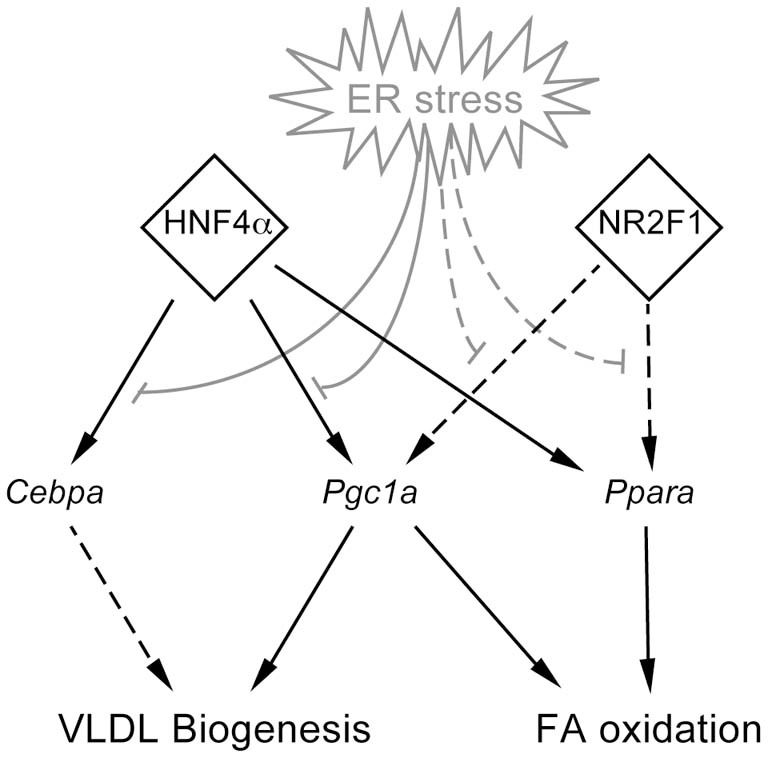
**Model for organization of lipid metabolic gene regulation during ER stress.** Experimentally demonstrated (here or elsewhere) relationships are shown using solid lines, while as-yet unvalidated relationships suggested by this work are shown by dashed lines.

With these facts in mind, we hypothesized that a “bottom-up” approach could be used to establish which ER stress-dependent metabolic gene expression changes occurred earliest, and to identify common regulators of those genes. To that end, our analysis predicted altered activity of HNF4α, which we then confirmed experimentally. We can conclude that its diminished binding to the promoters of *Cebpa* and *Pgc1a* is likely to contribute to the suppression of these genes, because knockdown of HNF4α is already known to have similar effects on metabolic genes to those reported here (Yin et al., 2011). We have combined the experimental and bioinformatic experiments described here with existing literature on the roles of HNF4α, C/EBPα, PGC-1α, and PPARα into a working model describing the genetic hierarchy of lipid metabolic gene regulation during stress (Figure 8). Although these relationships were elicited using TM, the observation that proteasome inhibition or overexpression of a misfolded secretory protein leads to similar genetic changes and lipid accumulation (Rutkowski et al., 2008), together with the fact that they were elicited using animals with a specific lesion in ER stress signaling, argue that they are likely to apply to ER stress in general.

Two key questions immediately arise for further study. The first of these is whether all of the metabolic gene regulatory changes downstream of ER stress are subordinate to HNF4α and subsequent changes in the expression of just one or a small number of rapidly suppressed master regulators such as *Cebpa*, *Pgc1a*, etc. Testing this hypothesis requires systematic overexpression of different metabolic transcriptional regulators, to see which blunt lipid accumulation during ER stress and how downstream metabolic genes respond. It is possible that HNF4α regulates both the proximal transcriptional regulators (C/EBPα, PGC-1α, etc.) and also genes encoding the downstream metabolic enzymes (CPT1A, ACOX1, etc.), which would provide a feedforward mechanism for suppression of these pathways. The second question is how ER stress influences HNF4α activity. Absent changes in HNF4α expression level or localization (Figure 7), the most likely scenarios are either covalent modification of HNF4α itself or modification, change in expression, or sequestration of a binding partner of HNF4α.

It is possible that the preponderance of bZIP factors produced during ER stress (ATF6α, ATF6β, ATF4, XBP1, and CHOP) could titrate a coregulator away from HNF4α. Persistent ER stress experienced in *Atf*6α^−/−^ animals causes prolonged UPR activation and expression of the other non-ATF6α UPR-regulated bZIPs (Rutkowski et al., 2008), which might therefore cause continued cofactor sequestration even as wild-type animals recover from stress. A sequestration model predicts that the strength and persistence of the stress will be a key factor in altering the activity of non-UPR transcription factors like HNF4α. Accordingly, chronic stresses would be expected to elicit different patterns of metabolic gene regulation than acute stresses, and strong stresses different patterns from milder ones. In support of this idea, exposure of zebrafish larvae to chronic but mild TM results in efficient induction of steatosis in the larval livers, and knockdown of ATF6α ameliorated this lipid accumulation. In contrast, a more acute but stronger ER stress—perhaps most akin to the exposures used in this work—led to less efficient steatosis that was exacerbated by ATF6α knockdown (Cinaroglu et al., 2011). The effects of stresses of different strengths and persistence have not yet been tested in a mammalian system, and will be important in validating or refuting the idea that pathophysiological conditions like obesity are in effect states of chronic ER stress. We also note that this work warrants an exploration of the roles of NR2F1 and ELK4 in hepatic gene expression. In fact, with all genes broken down into 81 possible expression profiles, we predict that other testable hidden regulatory nodes will emerge, and that searching these groups for conserved sequences will reveal nodes beyond the relatively small number that are linked to transcription factors in the JASPAR CORE database.

Finally, the scope of this work was limited to exploring the gene regulatory network linking acute ER stress to metabolism. Our gene expression results suggest that genetic suppression of VLDL production and fatty acid oxidation likely contribute to the steatotic phenotype while lipogenesis does not contribute, and might even be inhibited as a feedback mechanism to offset lipid accumulation. However, it remains to be determined whether these changes in mRNA expression are reflected in protein levels and consequent biochemical activities of the various pathways, and the extent to which they are also seen during physiological ER stresses such as obesity.

In summary, we have provided proof-of-principle that a bottom-up approach sheds light on the organization of metabolic gene regulation during ER stress and makes testable predictions about this organization. As a consequence, we have identified a novel regulatory node in the process. Beyond revealing a likely role for HNF4α in this regulation, it provides a resource for regulators of other coordinated gene expression groups to be discovered.

## Authors' contributions

AMA carried out qRT-PCR, ChIP, immunoblot, and bioinformatic analysis, and analyzed data. DDM carried out lipid analysis and immunohistochemistry, and analyzed data. RJK participated in the design and coordination of the microarray study. DTR conceived the study, participated in the design and coordination of all experiments, and drafted the manuscript. All authors read and approved the final manuscript.

### Conflict of interest statement

The authors declare that the research was conducted in the absence of any commercial or financial relationships that could be construed as a potential conflict of interest.

## Supplementary material

Figure S1 **Figure illustrates the gene expression profiles of lipid metabolic genes not part of Groups A–I**.

Table S1 **Table is a spreadsheet listing each of the probesets from the Affymetrix microarray used in this study, and the average expression values and group classification for each**.

Table S2 **Table lists the oligonucleotides used for qRT-PCR and ChIP analysis**.

## Abbreviations

bZIP: basic leucine zipper
ER: endoplasmic reticulum
TM: tunicamycin
UPR: unfolded protein response
VLDL: very low density lipoprotein.

## References

[B1] Arensdorf A. M. Rutkowski D. T. (2013). Endoplasmic reticulum stress impairs IL-4/IL-13 signaling through C/EBPβ-mediated transcriptional suppression. J. Cell. Sci. 126, 4026–4036 10.1242/jcs.130757 23813955 PMC3757336

[B2] Caviglia J. M. Gayet C. Ota T. Hernandez-Ono A. Conlon D. M. Jiang H. (2011). Different fatty acids inhibit apoB100 secretion by different pathways: unique roles for ER stress, ceramide, and autophagy. J. Lipid Res. 52, 1636–1651 10.1194/jlr.M016931 21719579 PMC3151684

[B3] Chen W. S. Manova K. Weinstein D. C. Duncan S. A. Plump A. S. Prezioso V. R. (1994). Disruption of the HNF-4 gene, expressed in visceral endoderm, leads to cell death in embryonic ectoderm and impaired gastrulation of mouse embryos. Genes Dev. 8, 2466–2477 10.1101/gad.8.20.2466 7958910

[B4] Chikka M. R. McCabe D. D. Tyra H. M. Rutkowski D. T. (2013). C/EBP homologous protein (CHOP) contributes to suppression of metabolic genes during ER stress in the liver. J. Biol. Chem. 288, 4405–4415 10.1074/jbc.M112.432344 23281479 PMC3567690

[B5] Cinaroglu A. Gao C. Imrie D. Sadler K. C. (2011). Activating transcription factor 6 plays protective and pathological roles in steatosis due to endoplasmic reticulum stress in zebrafish. Hepatology 54, 495–508 10.1002/hep.24396 21538441 PMC3145024

[B6] Dalton S. Treisman R. (1992). Characterization of SAP-1, a protein recruited by serum response factor to the c-fos serum response element. Cell 68, 597–612 10.1016/0092-8674(92)90194-H 1339307

[B7] Desvergne B. Michalik L. Wahli W. (2006). Transcriptional regulation of metabolism. Physiol. Rev. 86, 465–514 10.1152/physrev.00025.2005 16601267

[B8] Ellrott K. Yang C. Sladek F. M. Jiang T. (2002). Identifying transcription factor binding sites through Markov chain optimization. Bioinformatics 18 Suppl. 2, S100–S109 10.1093/bioinformatics/18.suppl_2.S100 12385991

[B9] Harding H. P. Zhang Y. Zeng H. Novoa I. Lu P. D. Calfon M. (2003). An integrated stress response regulates amino acid metabolism and resistance to oxidative stress. Mol. Cell 11, 619–633 10.1016/S1097-2765(03)00105-9 12667446

[B10] Hayhurst G. P. Lee Y. H. Lambert G. Ward J. M. Gonzalez F. J. (2001). Hepatocyte nuclear factor 4alpha (nuclear receptor 2A1) is essential for maintenance of hepatic gene expression and lipid homeostasis. Mol. Cell. Biol. 21, 1393–1403 10.1128/MCB.21.4.1393-1403.2001 11158324 PMC99591

[B11] Ho Sui S. J. Mortimer J. R. Arenillas D. J. Brumm J. Walsh C. J. Kennedy B. P. (2005). oPOSSUM: identification of over-represented transcription factor binding sites in co-expressed genes. Nucleic Acids Res. 33, 3154–3164 10.1093/nar/gki624 15933209 PMC1142402

[B12] Hollien J. Weissman J. S. (2006). Decay of endoplasmic reticulum-localized mRNAs during the unfolded protein response. Science 313, 104–107 10.1126/science.1129631 16825573

[B13] Janknecht R. Hunter T. (1997). Activation of the Sap-1a transcription factor by the c-Jun N-terminal kinase (JNK) mitogen-activated protein kinase. J. Biol. Chem. 272, 4219–4224 10.1074/jbc.272.7.4219 9020136

[B14] Jo H. Choe S. S. Shin K. C. Jang H. Lee J. H. Seong J. K. (2013). Endoplasmic reticulum stress induces hepatic steatosis via increased expression of the hepatic very low-density lipoprotein receptor. Hepatology 57, 1366–1377 10.1002/hep.26126 23152128

[B15] Kimura A. Nishiyori A. Murakami T. Tsukamoto T. Hata S. Osumi T. (1993). Chicken ovalbumin upstream promoter-transcription factor (COUP-TF) represses transcription from the promoter of the gene for ornithine transcarbamylase in a manner antagonistic to hepatocyte nuclear factor-4 (HNF-4). J. Biol. Chem. 268, 11125–11133 8496174

[B16] Kokame K. Kato H. Miyata T. (2001). Identification of ERSE-II, a new cis-acting element responsible for the ATF6-dependent mammalian unfolded protein response. J. Biol. Chem. 276, 9199–9205 10.1074/jbc.M010486200 11112790

[B17] Ktistaki E. Talianidis I. (1997). Chicken ovalbumin upstream promoter transcription factors act as auxiliary cofactors for hepatocyte nuclear factor 4 and enhance hepatic gene expression. Mol. Cell. Biol. 17, 2790–2797 9111350 10.1128/mcb.17.5.2790 PMC232130

[B18] Lee A. H. Iwakoshi N. N. Glimcher L. H. (2003). XBP-1 regulates a subset of endoplasmic reticulum resident chaperone genes in the unfolded protein response. Mol. Cell. Biol. 23, 7448–7459 10.1128/MCB.23.21.7448-7459.2003 14559994 PMC207643

[B19] Lee A. H. Scapa E. F. Cohen D. E. Glimcher L. H. (2008). Regulation of hepatic lipogenesis by the transcription factor XBP1. Science 320, 1492–1496 10.1126/science.1158042 18556558 PMC3620093

[B20] Li Y. Bevilacqua E. Chiribau C. B. Majumder M. Wang C. Croniger C. M. (2008). Differential control of the CCAAT/enhancer-binding protein beta (C/EBPbeta) products liver-enriched transcriptional activating protein (LAP) and liver-enriched transcriptional inhibitory protein (LIP) and the regulation of gene expression during the response to endoplasmic reticulum stress. J. Biol. Chem. 283, 22443–22456 10.1074/jbc.M801046200 18550528 PMC2504880

[B21] Lin J. Handschin C. Spiegelman B. M. (2005a). Metabolic control through the PGC-1 family of transcription coactivators. Cell Metab. 1, 361–370 10.1016/j.cmet.2005.05.004 16054085

[B22] Lin J. Yang R. Tarr P. T. Wu P. H. Handschin C. Li S. (2005b). Hyperlipidemic effects of dietary saturated fats mediated through PGC-1beta coactivation of SREBP. Cell 120, 261–273 10.1016/j.cell.2004.11.043 15680331

[B23] Lin J. Tarr P. T. Yang R. Rhee J. Puigserver P. Newgard C. B. (2003). PGC-1beta in the regulation of hepatic glucose and energy metabolism. J. Biol. Chem. 278, 30843–30848 10.1074/jbc.M303643200 12807885

[B24] Louet J. F. Hayhurst G. Gonzalez F. J. Girard J. Decaux J. F. (2002). The coactivator PGC-1 is involved in the regulation of the liver carnitine palmitoyltransferase I gene expression by cAMP in combination with HNF4 alpha and cAMP-response element-binding protein (CREB). J. Biol. Chem. 277, 37991–38000 10.1074/jbc.M205087200 12107181

[B25] Luebke-Wheeler J. Zhang K. Battle M. Si-Tayeb K. Garrison W. Chhinder S. (2008). Hepatocyte nuclear factor 4alpha is implicated in endoplasmic reticulum stress-induced acute phase response by regulating expression of cyclic adenosine monophosphate responsive element binding protein H. Hepatology 48, 1242–1250 10.1002/hep.22439 18704925 PMC2717709

[B26] Maere S. Heymans K. Kuiper M. (2005). BiNGO: a Cytoscape plugin to assess overrepresentation of gene ontology categories in biological networks. Bioinformatics 21, 3448–3449 10.1093/bioinformatics/bti551 15972284

[B27] Marciniak S. J. Yun C. Y. Oyadomari S. Novoa I. Zhang Y. Jungreis R. (2004). CHOP induces death by promoting protein synthesis and oxidation in the stressed endoplasmic reticulum. Genes Dev. 18, 3066–3077 10.1101/gad.1250704 15601821 PMC535917

[B28] Ota T. Gayet C. Ginsberg H. N. (2008). Inhibition of apolipoprotein B100 secretion by lipid-induced hepatic endoplasmic reticulum stress in rodents. J. Clin. Invest. 118, 316–332 10.1172/JCI32752 18060040 PMC2104481

[B29] Prifti E. Zucker J. D. Clement K. Henegar C. (2008). FunNet: an integrative tool for exploring transcriptional interactions. Bioinformatics 24, 2636–2638 10.1093/bioinformatics/btn492 18799481

[B30] Qiu Y. Pereira F. A. DeMayo F. J. Lydon J. P. Tsai S. Y. Tsai M. J. (1997). Null mutation of mCOUP-TFI results in defects in morphogenesis of the glossopharyngeal ganglion, axonal projection, and arborization. Genes Dev. 11, 1925–1937 10.1101/gad.11.15.1925 9271116 PMC316414

[B31] Rhee J. Ge H. Yang W. Fan M. Handschin C. Cooper M. (2006). Partnership of PGC-1alpha and HNF4alpha in the regulation of lipoprotein metabolism. J. Biol. Chem. 281, 14683–14690 10.1074/jbc.M512636200 16574644

[B32] Ron D. Habener J. F. (1992). CHOP, a novel developmentally regulated nuclear protein that dimerizes with transcription factors C/EBP and LAP and functions as a dominant-negative inhibitor of gene transcription. Genes Dev. 6, 439–453 10.1101/gad.6.3.439 1547942

[B33] Ron D. Walter P. (2007). Signal integration in the endoplasmic reticulum unfolded protein response. Nat. Rev. Mol. Cell Biol. 8, 519–529 10.1038/nrm2199 17565364

[B34] Rutkowski D. T. Arnold S. M. Miller C. N. Wu J. Li J. Gunnison K. M. (2006). Adaptation to ER stress is mediated by differential stabilities of pro-survival and pro-apoptotic mRNAs and proteins. PLoS Biol. 4:e374 10.1371/journal.pbio.0040374 17090218 PMC1634883

[B35] Rutkowski D. T. Wu J. Back S. H. Callaghan M. U. Ferris S. P. Iqbal J. (2008). UPR pathways combine to prevent hepatic steatosis caused by ER stress-mediated suppression of transcriptional master regulators. Dev. Cell 15, 829–840 10.1016/j.devcel.2008.10.015 19081072 PMC2923556

[B36] Schmidt D. Wilson M. D. Ballester B. Schwalie P. C. Brown G. D. Marshall A. (2010). Five-vertebrate ChIP-seq reveals the evolutionary dynamics of transcription factor binding. Science 328, 1036–1040 10.1126/science.1186176 20378774 PMC3008766

[B37] Schuldiner M. Weissman J. S. (2013). The contribution of systematic approaches to characterizing the proteins and functions of the endoplasmic reticulum. Cold Spring Harb. Perspect. Biol. 5:a013284 10.1101/cshperspect.a013284 23359093 PMC3578357

[B38] So J. S. Hur K. Y. Tarrio M. Ruda V. Frank-Kamenetsky M. Fitzgerald K. (2012). Silencing of lipid metabolism genes through IRE1alpha-mediated mRNA decay lowers plasma lipids in mice. Cell Metab. 16, 487–499 10.1016/j.cmet.2012.09.004 23040070 PMC3475419

[B39] Tyra H. M. Spitz D. R. Rutkowski D. T. (2012). Inhibition of fatty acid oxidation enhances oxidative protein folding and protects hepatocytes from endoplasmic reticulum stress. Mol. Biol. Cell 23, 811–819 10.1091/mbc.E11-12-1011 22262455 PMC3290641

[B40] Urano F. Wang X. Bertolotti A. Zhang Y. Chung P. Harding H. P. (2000). Coupling of stress in the ER to activation of JNK protein kinases by transmembrane protein kinase IRE1. Science 287, 664–666 10.1126/science.287.5453.664 10650002

[B41] Wang S. Chen Z. Lam V. Han J. Hassler J. Finck B. N. (2012). IRE1alpha-XBP1s induces PDI expression to increase MTP activity for hepatic VLDL assembly and lipid homeostasis. Cell Metab. 16, 473–486 10.1016/j.cmet.2012.09.003 23040069 PMC3569089

[B42] Wang Y. Vera L. Fischer W. H. Montminy M. (2009). The CREB coactivator CRTC2 links hepatic ER stress and fasting gluconeogenesis. Nature 460, 534–537 19543265 10.1038/nature08111 PMC2730924

[B43] Wolfrum C. Stoffel M. (2006). Coactivation of Foxa2 through Pgc-1beta promotes liver fatty acid oxidation and triglyceride/VLDL secretion. Cell Metab. 3, 99–110 10.1016/j.cmet.2006.01.001 16459311

[B44] Wu J. Rutkowski D. T. Dubois M. Swathirajan J. Saunders T. Wang J. (2007). ATF6alpha optimizes long-term endoplasmic reticulum function to protect cells from chronic stress. Dev. Cell 13, 351–364 10.1016/j.devcel.2007.07.005 17765679

[B45] Yamamoto K. Sato T. Matsui T. Sato M. Okada T. Yoshida H. (2007). Transcriptional induction of mammalian ER quality control proteins is mediated by single or combined action of ATF6alpha and XBP1. Dev. Cell 13, 365–376 10.1016/j.devcel.2007.07.018 17765680

[B46] Yamamoto K. Takahara K. Oyadomari S. Okada T. Sato T. Harada A. (2010). Induction of liver steatosis and lipid droplet formation in ATF6alpha-knockout mice burdened with pharmacological endoplasmic reticulum stress. Mol. Biol. Cell 21, 2975–2986 10.1091/mbc.E09-02-0133 20631254 PMC2929991

[B47] Yanai K. Hirota K. Taniguchi-Yanai K. Shigematsu Y. Shimamoto Y. Saito T. (1999). Regulated expression of human angiotensinogen gene by hepatocyte nuclear factor 4 and chicken ovalbumin upstream promoter-transcription factor. J. Biol. Chem. 274, 34605–34612 10.1074/jbc.274.49.34605 10574924

[B48] Yin L. Ma H. Ge X. Edwards P. A. Zhang Y. (2011). Hepatic hepatocyte nuclear factor 4alpha is essential for maintaining triglyceride and cholesterol homeostasis. Arterioscler. Thromb. Vasc. Biol. 31, 328–336 10.1161/ATVBAHA.110.217828 21071704 PMC3079249

[B49] Yoshida H. Okada T. Haze K. Yanagi H. Yura T. Negishi M. (2001). Endoplasmic reticulum stress-induced formation of transcription factor complex ERSF including NF-Y (CBF) and activating transcription factors 6alpha and 6beta that activates the mammalian unfolded protein response. Mol. Cell. Biol. 21, 1239–1248 10.1128/MCB.21.4.1239-1248.2001 11158310 PMC99577

[B50] Yoshida H. Okada T. Haze K. Yanagi H. Yura T. Negishi M. (2000). ATF6 activated by proteolysis binds in the presence of NF-Y (CBF) directly to the cis-acting element responsible for the mammalian unfolded protein response. Mol. Cell. Biol. 20, 6755–6767 10.1128/MCB.20.18.6755-6767.2000 10958673 PMC86199

[B51] Zhang C. Wang G. Zheng Z. Maddipati K. R. Zhang X. Dyson G. (2012). Endoplasmic reticulum-tethered transcription factor cAMP responsive element-binding protein, hepatocyte specific, regulates hepatic lipogenesis, fatty acid oxidation, and lipolysis upon metabolic stress in mice. Hepatology 55, 1070–1082 10.1002/hep.24783 22095841 PMC3319338

[B52] Zhang K. Shen X. Wu J. Sakaki K. Saunders T. Rutkowski D. T. (2006). Endoplasmic reticulum stress activates cleavage of CREBH to induce a systemic inflammatory response. Cell 124, 587–599 10.1016/j.cell.2005.11.040 16469704

[B53] Zhang K. Wang S. Malhotra J. Hassler J. R. Back S. H. Wang G. (2011). The unfolded protein response transducer IRE1alpha prevents ER stress-induced hepatic steatosis. EMBO J. 30, 1357–1375 10.1038/emboj.2011.52 21407177 PMC3094110

