# Supplementary material for: Temporal clustering of gene expression links the metabolic transcription factor HNF4α to the ER stress-dependent gene regulatory network
Source: Front Genet. 2013 Sep 24;4:188. doi: 10.3389/fgene.2013.00188 (PMC3781334; doi:10.3389/fgene.2013.00188)
Supplement: Figure S1 — Figure illustrates the gene expression profiles of lipid metabolic genes not part of Groups A–I. [file DataSheet2.PDF]

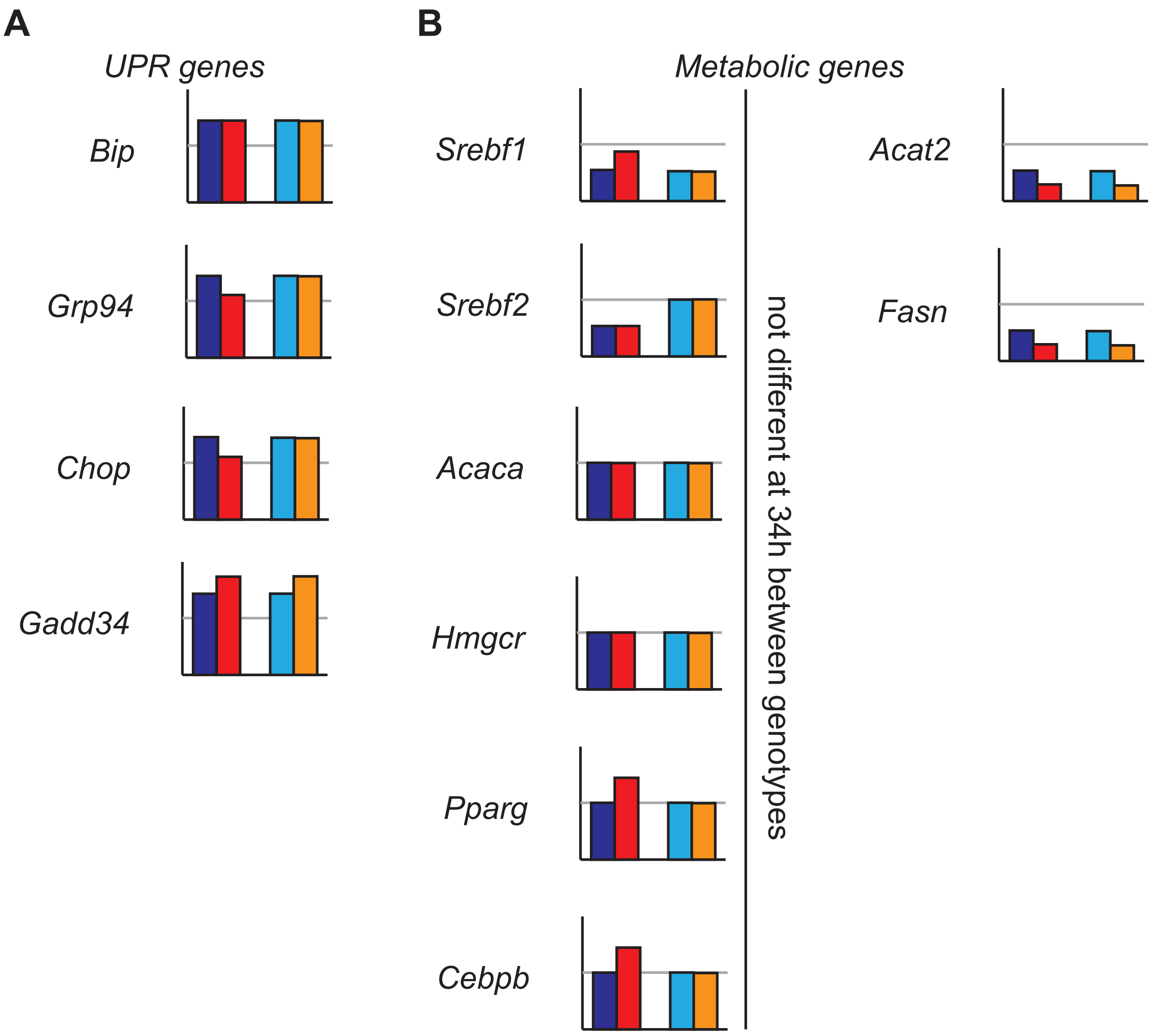

Figure S1. Expression profiles of genes not in groups A-I
(**A, B**) Each UPR (**A**) or metabolic (**B**) gene profiled by qRT-PCR in Figure 2 that was not a member of any defined group (A-I) is shown here.
